# Genetic Structure of Chinese Indigenous Goats and the Special Geographical Structure in the Southwest China as a Geographic Barrier Driving the Fragmentation of a Large Population

**DOI:** 10.1371/journal.pone.0094435

**Published:** 2014-04-09

**Authors:** Caihong Wei, Jian Lu, Lingyang Xu, Gang Liu, Zhigang Wang, Fuping Zhao, Li Zhang, Xu Han, Lixin Du, Chousheng Liu

**Affiliations:** 1 National Center for Molecular Genetics and Breeding of Animal, Institute of Animal Sciences, Chinese Academy of Agricultural Sciences, Beijing, People’s Republic of China; 2 National Center of Preservation & Utilization of Genetic Resources of Animal, National Animal Husbandry Service, Beijing, People’s Republic of China; China Agricultrual University, China

## Abstract

**Background:**

China has numerous native domestic goat breeds, however, extensive studies are focused on the genetic diversity within the fewer breeds and limited regions, the population demograogic history and origin of Chinese goats are still unclear. The roles of geographical structure have not been analyzed in Chinese goat domestic process. In this study, the genetic relationships of Chinese indigenous goat populations were evaluated using 30 microsatellite markers.

**Methodology/Principal Findings:**

Forty Chinese indigenous populations containing 2078 goats were sampled from different geographic regions of China. Moderate genetic diversity at the population level (H_S_ of 0.644) and high population diversity at the species level (H_T_ value of 0.737) were estimated. Significant moderate population differentiation was detected (F_ST_ value of 0.129). Significant excess homozygosity (F_IS_ of 0.105) and recent population bottlenecks were detected in thirty-six populations. Neighbour-joining tree, principal components analysis and Bayesian clusters all revealed that Chinese goat populations could be subdivided into at least four genetic clusters: Southwest China, South China, Northwest China and East China. It was observed that the genetic diversity of Northern China goats was highest among these clusters. The results here suggested that the goat populations in Southwest China might be the earliest domestic goats in China.

**Conclusions/Significance:**

Our results suggested that the current genetic structure of Chinese goats were resulted from the special geographical structure, especially in the Western China, and the Western goat populations had been separated by the geographic structure (Hengduan Mountains and Qinling Mountains-Huaihe River Line) into two clusters: the Southwest and Northwest. It also indicated that the current genetic structure was caused by the geographical origin mainly, in close accordance with the human’s migration history throughout China. This study provides a fundamental genetic profile for the conservation of these populations and better to understand the domestication process and origin of Chinese goats.

## Introduction

The archaeological and genetic evidence suggests that goat (Capra hircus) was probably first domesticated in the Fertile Crescent region of the Near East ≈10,000 years ago [1], [2]. The domestic goat has always played an important role in providing a full range of useful products to human society, such as meat, milk, pelts and fiber, especially in China and other development countries [3]. Goats are the most adaptable and geographically wide spread livestock species, ranging from the mountains of Siberia to the deserts and reopics of Africa [4], [5]. To date, there are about 1,000 goat breeds, and more than 875 million goats are kept around the world, according to the statistics of the UN Food and Agriculture Organization (http://www.fao.org/corp/statistics/en/). In China, there are about 58 native domestic goat breeds, distributed in different environmental areas including the north pastoral region, the Qinghai-Tibet plateau region, the mixed pastoral-agricultural region, and the north and south agricultural region (Animal Genetic Resources in China: Sheep and Goats, 2011). During this decade, these domestic breeds represent important genetic resources and have been aroused us more great attention, therefore, conservation of the domestic animal diversity is essential to meet the future needs. Considering the special economic and ecological characteristics, China recently has become concerned about these indigenous animal populations and initiated the conservation programs, such as setting the conservation area, conservation farm and the genebank of genetic resource for special breeds.

The study of genetic diversity within and across breeds provides insight into population structure and relationships, and is essential for the conservation of these indigenous breeds [6], [7]. Studies on the genetic diversity of domestic goats using mitochondrial DNA, microsatellites and single nucleotide polynorphism (SNP) markers have been conducted over few years in the world [4], [8], [9], [10], [11]. These studies have demonstrated that the domestic goat showed a weak phylogeographic structure. Recently, Kijas et al also used the goat genome-wide SNP array to assess the genetic diversity and polledness in divergent goat populations [12]. It is suggested that the insight on breed development and introgression provided by the different types of genetic markers is complementary. The nuclear microsatellite genetic markers have been extensively used to assess genetic diversity and inbreeding levels of populations, introgression from other genetic groups, genetic differentiation and population structure, even reflect the consequences of genetic drift, founder effects and population admixture [9], [13], [14], [15].

In the Western region of China, the Hengduan Mountains, as a natural geographical barrier, block the Qinghai-Tibet Plateau and the Yunnan-Guizhou Plateau, and have the most complex river systems and a profoundly complex and dynamic geological history [16]. In addition, the Qinling Mountains represent an important geographic barrier in Eastern Asia that divides the current mainland of China into southern and northern and temperate and semi-tropical regions [17]. These mountains had divided the West China into Northwest China and Southwest China, and results to the different climatic characteristic and geographical structure.

Since China is a vast subcontinent with the complex geographical situation, Chinese goat breeds exhibit enormous variations in fecundity; production of meat, milk, and fiber; draughtability; disease resistance; and heat tolerance. In addition, many previous studies of Chinese goats were conducted in the limited number of samples within fewer breeds and regions [8], [9], [15], [18], [19]. The objective of the present study was to use 30 nuclear microsatellite markers to analyze the genetic relationships and genetic differentiation of 40 Chinese indigenous populations containing 2078 goats sampled from different geographic regions of China. Furthermore, the bottleneck, the origins and evolutionary trajectories of Chinese domestic goats also had been investigated. Our results demonstrated that Chinese goat populations could be subdivided into at least the following four genetic clusters: Southwest China, South China, Northwest China and East China, and the genetic diversity of Northern China goats was highest among these clusters. It was also suggested that the current genetic structure of Chinese goats was caused by the geographical origin mainly, in close accordance with the human’s migration history throughout China. This information may be valuable for the conservation and maintaining diversity and genetic uniqueness of the genetic resource of these indigenous goat breeds, is better to understand the domestication process and origin of Chinese goats.

## Materials and Methods

### Biological Samples Collection

**Figure 1:**
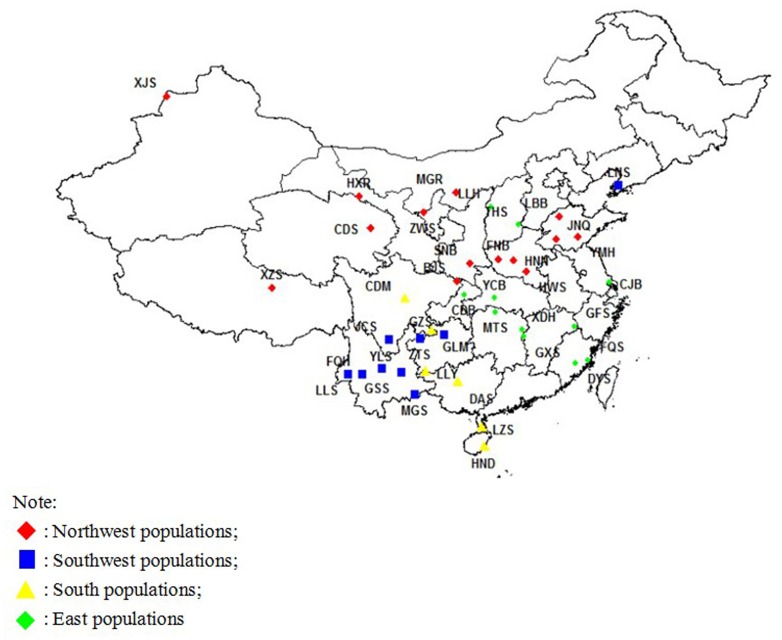
Geographic distribution of the 40 goat populations in China.

In total, 2078 animals representing 40 goat breeds were studied (Additional file: Table S1). Goats were sampled from 23 provinces or autonomous regions in China, including almost all of the geographical regions, from high latitude to plains (Figure 1). The sampling protocol for each goat breed was below: coming from the main producing area, meeting the characteristics and features of the breed, without relationship in the three generations, healthy, 30 males and 30 females. The blood samples were collected by the owner of the animal or a collaborator and shipped to this laboratory for processing. All experimental procedures were approved by the Law of Animal Husbandry in People’s Republic of China (Dec 29, 2005). The whole study protocols for collection of the blood samples of experimental individuals were reviewed and approved by the Biological Studies Animal Care and Use Committee of National Animal Husbandry Service, Beijing, People’s Republic of China. The genomic DNA was isolated from cryopreserved blood samples using DNeasy Blood & Tissue Kit (Qiagen, Valencia, CA). After removing the poor quality DNA samples, the number of samples in this study was 2078 finally.

### Microsatellite Markers

A common set of 30 microsatellites were selected from a panel of 31 markers recommended for genetic diversity studies by the International society for Animal Genetics (ISAG)/Food and Agriculture Organization of the United Nations (FAO) working group: CSRD247, ETH10, MAF209, OARAE54, SRCRSP15, SRCRSP3, SRCRSP5, TGLA53, DRBP1, ILSTS087, INRABERN172, MAF065, MCM527, OARFCB20, SPS113, SRCRSP8, ILSTS029, INRA023, INRA063, INRABERN185, P19(DYA), SRCRSP23, SRCRSP9, TCRVB6, BM6444, ILSTS011, ILSTS005, SRCRSP7, OARFCB48, MAF70.

The 30 microsatellite markers were amplified in multiplex polymerase chain reactions (PCRs) using fluorescence-labelled primers. PCR products had been run on PAGE-SDS gel to check the real size, and separated using an ABI-PRISM 3100-Avant Genetic Analyzer (Applied Biosystems, Fster City, CA) according to manufacturer recommendations. Allele sizes relative to an internal size standard (GS400HD) were determined using GENEMAPPER 3.5 (Applied Biosystems, Fster City, CA).

### Statistical Analysis

For each microsatellite locus, the mean number of alleles, observed heterozygosities (H_O_), expected (H_E_) heterozygosities average genetic diversity (H_S_) both within populations and among populations and total gene diversity (H_T_) were obtained from the FSTAT 2.9.3 software [20]. Wright’s F-statistics were estimated from FSTAT 2.9.3 using Weir and Cockerham method [21]. Deviations from Hardy-Weinberg equilibrium (HWE) were assessed with GENEPOP v 4.2 software [22]. Unbiased estimates of exact P-values were obtained using the Markov Chain Monte Carlo algorithm of 10000 dememorization steps, 100 batches, and 5000 iterations. The linkage disequilibrium between each pair of loci was assessed from GENEPOP software [23].

To estimate whether of the reduction in the effective population size, the Wilcoxon’s signed-rank tests (one-tailed) with the null hypothesis, H_E_>H_EQ_, and the alternative hypothesis, H_E_<H_EQ_, were used under the assumption of mutation-drift equilibrium in the infinite-alleles model (IAM), two-phase model of mutation (TPM) and the stepwise mutation model (SMM), and the allele frequency distribution mode shift analysis were performed using BOTTLENECK version 1.2.02 [24].

According to the geographic origin of these populations, a hierarchical analysis of variance was performed to partition the total genetic variance into components due to within-individuals, among-populations and among individuals within populations’ differences. Variance components were used to compute the fixation indexes and their significance was tested using a non-parametric permutation approach [25]. The analysis of molecular variance (AMOVA) module of ARLEQUIN 3.5.1.2 software was used to perform the computation [26].

To illustrate the genetic divergence among the breeds, the Reynolds distances were estimated by the PHYLIP package to reconstruct the neighbor-joining consensus tree, with the bootstrapping of 1,000 replicates, and the dendrogram was depicted using the software package TreeView version 1.4.0 [27], [28]. Additionally, the pattern of genetic variation among populations was also assessed by principal components analysis (PCA) which was based on allele frequencies using the Multivariate Statistical Package (MVSP) version 3.13b (Kovach Computing Services, Anglesey, Wales, UK).

To study population genetic structure and to detect the most likely number of clusters (K) in the data set, the STRUCTURE 2.3 software was performed [29]. The Structure algorithm assumes K populations, each of which is in Hardy-Weinberg and linkage equilibrium and characterized by a set of allele frequencies at each locus. To choose the appropriate number of inferred clusters to model the data, 2 to 40 inferred clusters were performed with 10 independent runs each. All analyses were performed with a burn-in length of 100,000 followed by 100,000 Markov Chain Monte Carlo iterations for each of K. To find optimal alignments of independent runs, the computer program CLUMPP 1.1 was used; and the output obtained was used directly as input by the cluster visualization program Distruct 1.1 software [30], [31].

## Results

### Genetic Diversity

In the present study, a set of 30 microsatellite markers listed in Table 1 was used to analysis samples of the 40 goat populations (Table S1). The result of microsatellite markers amplification analyze showed that the total number of alleles was 471, and the largest number of alleles was found at locus BM6444 (35) and the smallest at locus MAF 209 (4), with an average of 15.7 alleles per locus (Table 1). The average genetic diversity (H_S_) among the locus was 0.644 and ranging from 0.237 to 0.820. Additionally, the average overall genetic diversity (H_T_) estimate among the populations was 0.737, ranging from 0.274 to 0.907. As shown in Table 2, the genetic variability of each population was studied. The highest mean number of alleles (MNA) was 8.27 in the FNB goat and the lowest was 4.33 in the GFS goat. Among these populations, the allelic richness (AR) based on 23 diploid individuals per population ranged from 3.89 in DYS goat to 7.35 in FNB. The estimated H_E_ per population ranged from 0.5070 in DYS to 0.7378 in FNB. H_O_ ranged from 0.4336 in DYS to 0.6730 in HNN. It is observed that the FNB population was the highest one in MNA, AR and H_E_ among all the 40 populations, showing the higher genetic variation. In contrast, the DYS population showed the lower genetic variation than other populations. Additionally, it is obtained that H_O_ was lower than H_E_ in all the populations analyzes in this study. As shown in Table 2, the F_IS_ over all the loci was found to be significant in thirty-eight populations except CDM and LLH, whereas the F_IS_ within populations was significant for twenty loci (CSRD247, OARAE54, SRCRSP15, SRCRSP3, SRCRSP5, TGLA53, DRBP1, ILSTS087, INRABERN172, MAF065, OARFCB20, SPS113, SRCRSP8, INRA023, INRA063, P19(DYA), TCRVB6, BM6444, ILSTS011, SRCRSP7) (Table 1). These results suggested a significant deviation from the Hardy-Weinberg equilibrium.

**Table 1:** Genetic diversity measures estimated at each 30 loci across the 40 Chinese indigenous goat populations.

Locus	Location	TA	Allele size	H_O_	H_S_	H_T_	F_IT_	F_ST_	F_IS_
CSRD247	14	13	220–247	0.667	0.723	0.804	0.174***	0.105***	0.078***
ETH10	5	11	200–210	0.376	0.309	0.379	0.012	0.189***	−0.218
MAF209	17	4	100–104	0.246	0.237	0.274	0.104***	0.138***	−0.039
OARAE54	25	15	115–138	0.582	0.607	0.722	0.200***	0.161***	0.047***
SRCRSP15	unknown	11	172–198	0.507	0.542	0.626	0.192***	0.134***	0.067***
SRCRSP3	10	10	109–123	0.527	0.594	0.714	0.260***	0.167***	0.111***
SRCRSP5	21	14	156–178	0.670	0.693	0.786	0.146***	0.121***	0.029**
TGLA53	16	18	126–160	0.358	0.622	0.703	0.503***	0.116***	0.438***
DRBP1	23	24	195–229	0.358	0.758	0.838	0.574***	0.097***	0.528***
ILSTS087	6	13	135–155	0.702	0.764	0.853	0.182***	0.105***	0.085***
INRABERN172	26	12	234–256	0.570	0.607	0.650	0.123***	0.066***	0.061***
MAF065	15	18	116–158	0.709	0.733	0.792	0.103***	0.074***	0.031**
MCM527	5	12	165–187	0.603	0.610	0.669	0.097***	0.088***	0.010
OARFCB20	2	15	93–112	0.589	0.652	0.746	0.208***	0.132***	0.088***
SPS113	10	12	134–158	0.671	0.697	0.768	0.129***	0.096***	0.037**
SRCRSP8	unknown	16	215–255	0.560	0.743	0.830	0.314***	0.104***	0.235***
ILSTS029	3	20	148–170	0.661	0.653	0.776	0.156***	0.163***	−0.009
INRA023	3	19	196–215	0.489	0.635	0.863	0.437***	0.273***	0.226***
INRA063	18	23	164–186	0.462	0.663	0.828	0.438***	0.205***	0.293***
INRABERN185	18	18	261–289	0.504	0.514	0.674	0.256***	0.244***	0.016
P19(DYA)	23	18	160–196	0.636	0.690	0.771	0.178***	0.105***	0.081***
SRCRSP23	unknown	18	81–119	0.714	0.694	0.811	0.117***	0.146***	−0.034
SRCRSP9	12	17	99–135	0.747	0.743	0.839	0.115***	0.118***	−0.003
TCRVB6	10	24	217–255	0.590	0.722	0.797	0.263***	0.098***	0.183***
BM6444	2	35	118–200	0.541	0.820	0.907	0.411***	0.099***	0.347***
ILSTS011	14	13	256–294	0.648	0.675	0.747	0.126***	0.098***	0.031*
ILSTS005	10	13	172–218	0.657	0.614	0.704	0.071***	0.130***	−0.068
SRCRSP7	6	7	117–131	0.493	0.569	0.642	0.237***	0.116***	0.137***
OARFCB48	17	12	149–173	0.731	0.715	0.786	0.067***	0.092***	−0.027
MAF70	4	14	134–168	0.708	0.722	0.807	0.124***	0.109***	0.017
	All	471		0.576	0.644	0.737	0.220***	0.129***	0.105***

Note: *Location*, the locus located on the chromosomes; *T_A_*, total number of alleles; *H_O_*, observed heterozygosity; *H_S_*, gene diversity; *H_T_*, overall gene diversity; *F_IS_*, *F_ST_* and *F_IT_*, measures of the F-statistics.

*P<0.05;

**P<0.01;

***P<0.001.

**Table 2:** Genetic diversity measures estimated using 30 microsatellite loci in each of 40 goat populations.

No.	Breed	n	TNA	MNA	AR	H_E_	H_O_	F_IS_
1	LLS	52	180	6.00	5.21	0.5882	0.4908	0.167
2	MGS	50	160	5.33	4.68	0.5981	0.5729	0.043
3	YLS	47	180	6.00	5.36	0.6283	0.5517	0.123
4	ZTS	50	178	5.93	5.16	0.5925	0.5299	0.107
5	JCS	60	172	5.73	4.95	0.6000	0.5754	0.041
6	GSS	59	230	7.67	6.56	0.6797	0.5490	0.194
7	FQH	43	137	4.57	4.23	0.5584	0.5178	0.073
8	GZS	50	169	5.63	4.91	0.5823	0.5465	0.062
9	LNS	58	193	6.43	5.69	0.6635	0.6198	0.066
10	CDM	58	178	5.93	5.09	0.6060	0.5952	0.018
11	GLM	60	195	6.50	5.66	0.6492	0.5965	0.082
12	LLY	52	169	5.63	5.13	0.6395	0.6003	0.062
13	LZS	56	164	5.47	4.71	0.5826	0.5393	0.075
14	HND	58	178	5.93	5.18	0.5944	0.5078	0.147
15	DAS	36	178	5.93	5.60	0.6601	0.5574	0.157
16	MGR	48	208	6.93	6.19	0.6614	0.5556	0.161
17	CDS	59	237	7.90	6.87	0.7090	0.6530	0.080
18	XJS	59	247	8.23	7.03	0.6891	0.6155	0.108
19	XZS	53	221	7.37	6.60	0.6846	0.6116	0.108
20	HXR	43	243	8.10	7.27	0.7171	0.6339	0.117
21	ZWS	25	206	6.87	6.80	0.6941	0.6218	0.106
22	SNB	56	194	6.47	5.83	0.6830	0.6010	0.121
23	BJS	58	202	5.73	5.70	0.6815	0.5532	0.190
24	FNB	45	248	8.27	7.35	0.7378	0.6186	0.163
25	HNN	60	220	7.33	6.31	0.7076	0.6730	0.049
26	HWS	50	221	7.37	6.75	0.7294	0.5882	0.195
27	JNQ	59	219	7.30	6.56	0.7369	0.6461	0.124
28	YMH	53	235	7.83	6.73	0.7043	0.5573	0.210
29	LBB	48	230	7.67	6.72	0.7069	0.6279	0.113
30	LLH	58	193	6.43	5.52	0.6618	0.6476	0.022
31	THS	60	194	6.47	5.59	0.6569	0.6151	0.064
32	CJB	42	173	5.77	5.32	0.6440	0.6234	0.032
33	CDB	60	194	6.47	5.39	0.6273	0.5536	0.118
34	GFS	44	130	4.33	4.02	0.5269	0.5051	0.042
35	GXS	43	144	4.80	4.35	0.5836	0.5375	0.080
36	MTS	60	161	5.37	4.78	0.6251	0.5689	0.091
37	YCB	52	178	5.93	5.19	0.6549	0.6227	0.050
38	XDH	52	161	5.37	4.74	0.6080	0.5493	0.097
39	FQS	55	147	4.90	4.30	0.5692	0.4745	0.168
40	DYS	47	132	4.40	3.89	0.5070	0.4336	0.146
	Average		190	6.31	5.60	0.6433	0.5760	

*Note: n*, sample size; *TNA*, total number alleles; *MNA*, mean number of alleles; *AR*, allelic richness, *H_E_*, heterozygosity estimates, *H_O_*, heterozygosity observed; *F_IS_*, fixation index.

According to Wilcoxon’s signed-rank test performed using BOTTLENECK, thirty-six populations (LLS, MGS, YLS, ZTS, JCS, GSS, FQH, GZS, LNS, CDM, GLM, LLY, LZS, HND, DAS, MGR, XZS, HXR, ZWS, SNB, FNB, HNN, HWS, YMH, LBB, LLH, THS, CJB, CDB, GFS, GXS, MTS, YCB, XDH, FQS, DYS) showed a significant deviation (excess heterozygosity) from the mutation-drift equilibrium under the SMM (after sequential Bonferroni correction), whereas under the IAM and TPM, all the 40 goat populations showed a significant deviation from the mutation-drift equilibrium, suggesting that decreased in the population size had occurred due to a bottleneck effect (Table S2). The mode shift test also detected that 26 populations showed the distortion of allele frequency, whereas another 14 populations showed a typical property of a population in equilibrium were ZTS, GSS, GZS, GLM, CDS, XJS, BJS, HNN, HWS, JNQ, YMH, LBB, LLH and CDB (Table S2).

### Genetic Differentiation

As shown in Table 1, the population differentiation was significant within each locus, with the average F_ST_ value of 0.129 detected, ranging from 0.066 (INRABERN172) to 0.273 (INRA023). The measure of the relative genetic differentiation for the pairwise F_ST_ values between populations was found to be significant (*P*<0.05, Table S3), except for four pairwise populations (YLS-ZTS, CDS-XJS, ZWS-HXR and YCB-MTS).

F-statistics and their 95% confidence intervals obtained with 10000 bootstraps over 30 loci were as follow: F_IS_ = 0.105 (−0.007–0.552), F_IT_ = 0.223 (−0.028–0.601) and F_ST_ = 0.132 (0.072–0.293). Breeds estimated of F_IS_ were computed using FSTAT (Goudet, 2000) and ranged from 0.018 to 0.21 (Table 1). Breeds with the highest F_IS_ values were YMH, HWS, GSS and BJS. The breeds with the lowest F_IS_ value were CDM, LLH, JCS and MGS.

The AMOVA results indicated that 77.72% of the total variance was partitioned within individuals, whereas 13.15% and 9.12% of the total variance were explained by differences among the populations and among the individuals within populations, respectively (*P*<0.001, Table 3).

**Table 3:** Results of analysis of molecular variance (AMOVA) for 40 populations of Chinese goats.

Source of variation	d.f	Sum of squares	Variation components	Variations (%)
Among populations	40	6405.542	1.46086	13.15127***
Among individuals within populations	2038	21923.663	1.01360	9.12486***
Within individuals	2073	18106	8.63368	77.72387***

d.f., degrees of freedom.

***P<0.001, *P*-values were obtained by 20000 permutations.

### Population Structure

**Figure 2:**
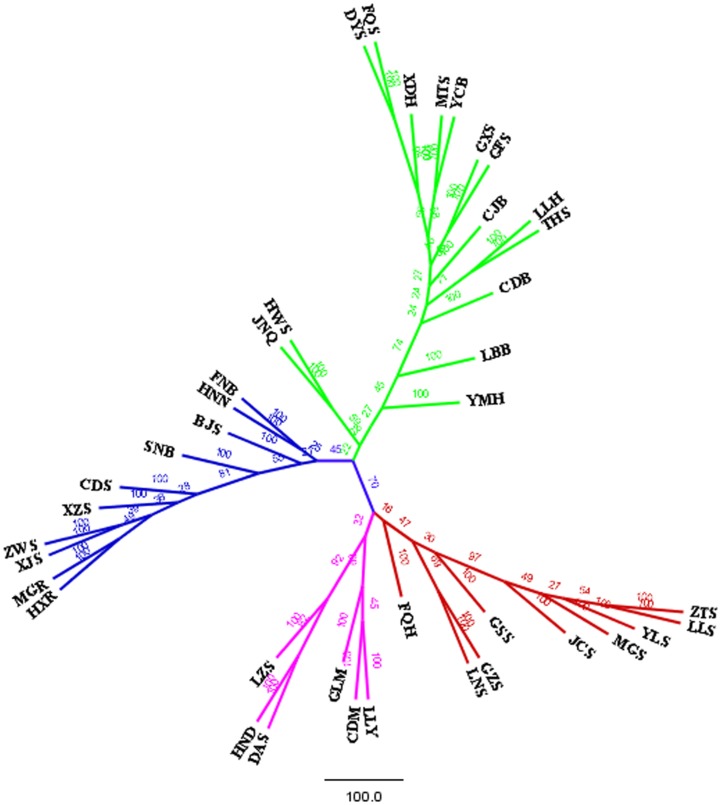
The neighbor-joining tree of 40 goat populations in China was constructed based on Nei’s genetic distance (D_A_). The population abbreviations are shown in Table 1. The numbers on the nodes indicate the bootstrap values (%) obtained from 1000 replications.

The neighbor-joining tree constructed on the Reynolds distances was used to portray the degree of the genetic relationships among the goat populations. The tree showed that the 40 populations were clustered into four major clades in China (Figure 2). As shown in the Figure 2, the Southwest China clade comprised ZTS, LLS, YLS, MGS, JCS, GSS, GZS, LNS and FQH, whereas the South China clade comprised LLY, CDM, GLM, DAS, HND and LZS. Similarly, the Northwest China clade comprised HXR, MGR, XJS, ZWS, XZS, CDS, SNB, BJS, HNN, FNB, JNQ and HWS, whereas the East China comprised YMH, LBB, CDB, THS, LLH, CJB, GFS, GXS, YCB, MTS, XDH, FQS and DYS. As shown in the tree, it was suggested that each population had been clustered to its geographical distribution and origin (Figure 1), not to the administrative division of China. For example, the population JCS was located in the Hengduan mountains, south of Sichuan province, therefore, which had divided into the Southwest cluster. Similar situation was also happened in BJS which is located in the northeast of Sichuan province, nearby the Qingling Mountains, but had been divided into the Northwest cluster, not to the Southwest cluster or South cluster. It was also observed that the populations (YMH, LBB, JNQ and HWS) in the Shandong province and Henan province located in the North China Plain had been grouped to the East China cluster, but admixed with the North China cluster. The cause of this situation maybe is that there is no mountain to restrict the gene flow between the two clusters: East China and Northwest China.

**Figure 3:**
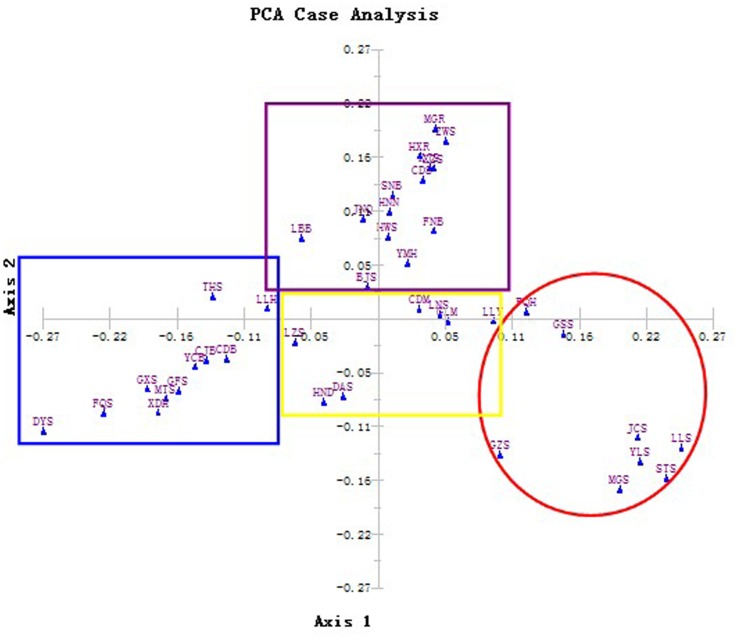
PCA analysis.

The population-based PCA was performed using allele frequencies of the 30 microsatellite markers in 40 goat populations. The first principal component accounts for 21.09% of the total variation and especially separated the whole populations into the West and the East. The second principal component condenses 13.42% of the variation, which separated the 40 populations into the South and North. The PCA clustered these populations into four groups, as shown in the Figure 3, which corresponded to the four gene pools (Figure 2). However, there was some ambiguity regarding the placement of the individuals of populations LBB, JNQ, YMH, HHS and LNS, as the individuals of these populations represented a mixture of the other groups. The results are mostly coincided with the topology of the neighbor-joining tree. The populations are grouped on the basis of geographical distribution and origin generally.

**Figure 4:**
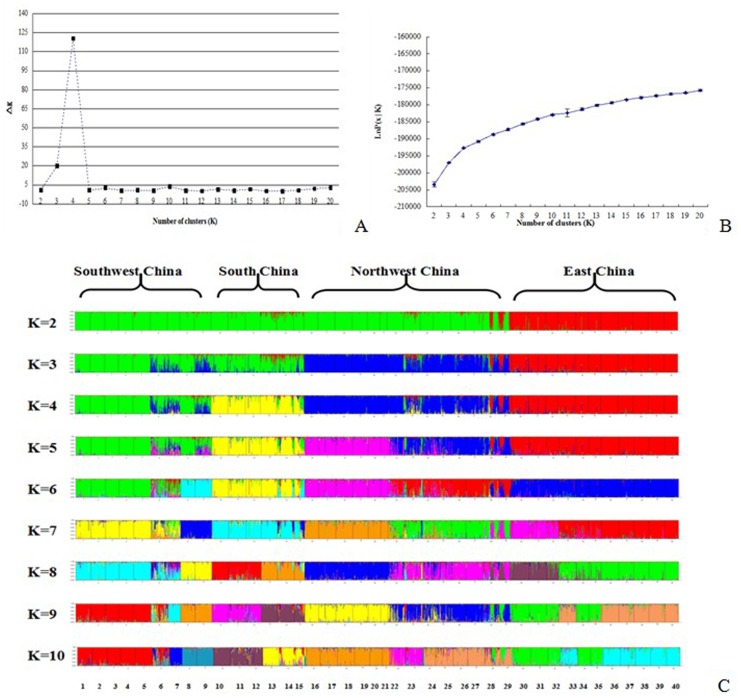
Population structure of 40 goat breeds based on 30 microsatellite loci using STRUCTURE. *K* was estimated using (A) the posterior probability of the data given each *K* (10 replicates) and (B) the distribution of Δ*K*, and (C) the colored clusters represented *K* from 2 to 10 were detected from STRUCTURE analysis. The number given below was corresponding to the name of each breed (seen in the Table 2).

The Bayesian clustering model-based method allowed for assessment of the genetic structure and admixture among the populations. The results of Bayesian-based clustering revealed that the inference of the number of gene pools (*K*) was not straightforward because log-likelihood values for the data conditional on *K*, and lnP(x|*K*), increased progressively as *K* increased, as shown in Figure 4. When the number of populations varied from *K* = 2 to 40, the largest change in the log of the likelihood function (△*K*) was when *K* = 4. The △*K* value obtained was 19.90 at *K* = 3, 120.50 at *K* = 4, 0.78 at *K* = 5, 2.59 at *K* = 6, and 0.52 at *K* = 7. Changing the assumptions of an “equal alpha for each population” and “correlated allele frequencies” could not change the final result. The proportion of individuals in each population a*s*signed into four clusters (or gene pools) is shown in Figure 4, and the corresponding 40 populations were plotted onto the map, as shown in Figure 1. The results of Bayesian clustering for *K* = 2 indicated that there was a clear transition from the West China populations (green) to the East China populations (red). At *K* = 3, the West China populations had been divided into two clusters, the Southwest China populations (green) and the Northwest China populations (blue). At *K* = 4, the △*K* value reached a maximum, and the Southwest China populations subsequently had been divided into the Southwest China populations (green) and South China populations (yellow). It was suggested that gene pool 1(Southwest China) included nine populations: LLS, MGS, YLS, ZTS, JCS, GSS, FQH, GZS and LNS, and that this pool was most abundant in the Yunnan-Guizhou Plateau, except LNS in the Liaoning Province. Similar results were also found in the NJ-tree and PCA analysis. The gene pool 2 (South China) surrounded the Yunnan-Guizhou Plateau and reached the southernmost of China along the Pearl River, and that this pool included six populations: CDM, GLM, LLY, LZS, HND and DAS. Gene pool 3(Northwest China) was predominant on the northern of Qingling Mountains –Huaihe River Line which represented the geographical boundaries of North and South in China, especially Eastern China, and encompassed fourteen populations: MGR, CDS, XJS, XZS, HXR, ZWS, SNB, BJS, FNB, HNN, HWS, JNQ, YMH and LBB. Gene pool 4 (East China) was predominant on Yangtze River hills and encompassed twelve populations: LLH, THS, CJB, CDB, GFS, GXS, MTS, YCB, XDH, FQS and DYS. However, the populations: YMH and LBB, which located at the Shandong Province, showed a mixture of the four ancestral genetic resources (Figure 1 and Figure 4). With the exception of LNS, it was obvious that the four gene pools were almost in accordance with the natural distribution of goat in China. The three clustering results from the three different analysis methods were clearly in total agreement with each other.

## Discussion

### Moderate Genetic Diversity and High Genetic Differentiation of the Goat Populations

In the current study, we attempted to estimate the genetic diversity and differentiation of the 40 China indigenous goat populations using 30 microsatellite markers. It was detected that the moderate genetic diversity at the population level (H_S_ = 0.644, H_E_ = 0.6433) and high population diversity (H_T_ = 0.737) at the species level. Our results showed that the genetic variation estimated by MNA and AR in Chinese indigenous populations was lower than that of European breeds which was 5.2–9.1 MNAs, with an average of 7.1 and 6.1–7.9 AR values, but was higher than that of EastAsian indigenous populations which was 2.5–7.6, with an average of 5.8 and 2.3–6.89 AR values [10], [32]. In the study of Nomura et al, the genetic diversity of the populations in Mongolia and Bangladesh was higher than that of other countries, such as Japan, Korea and Indonesia [32]. Similar result also occurred in our study. In this investigation, genetic diversity of the goats in the Northwest populations (such as XJS, MGR and FNB goat) was comparatively higher than that in others clusters. Although, the populations were different, the similar result also occurred in the study of Li et al, that the diversity of the North China populations, such as Taihang goat and Neimonggol goat, was also higher than that of other populations (such as Small-xiang goat, Chuangdong White goat and Nanjiang Brown goat) [9]. The reason of the high genetic diversity maybe is that the Northwest China populations are nearby the center of Fertile Crescent region of Near East, and the gene flow was converged in the region.

Significant population differentiation was indicated by the high F_ST_ values (averaging 0.129), similar to the EastAsian indigenous populations with the mean value of 0.13, but higher than that of the European breeds, which had a mean value of 0.07 [10], [32]. The Eastern China populations showed the higher pairwise F_ST_ values than the mean, suggesting that these goat populations showed higher genetic differentiation than other populations. The regions of these Eastern populations were located farthest from the goat domestication center of West Asia.

In the present study, Bottlenecks occurred in thirty-six populations, except CDS, XJS, BJS and YMH, which were located in North of Qinling-Huaihe Line. Our results indicated that population bottleneck had happened over the entire distribution of Chinese goats. It is the first report to analysis the bottleneck in Chinese indigenous goats using 30 microsatellite markers. The results of Bottleneck analysis of Chinese goats would help us to understand the developing progress of these populations, and provide new sights to establish the conservation programs to maintain the high genetic diversity of these indigenous goat populations.

In the present study, the results of the F_IS_ value over all the loci (Table 2) and the F_IS_ within individual populations per locus (Table 1) indicated that there was a significant deviation from the Hardy-Weinberg (HW) equilibrium in the 40 goat populations. The main reasons of the significant positive values of the F_IS_ are the existence of inbreeding and excess homozygosity.

### Distinct Genetic Structure of the Chinese Goat Populations

According to the results of Bayesian model-based clustering and the NJ tree based on Nei’s genetic distance, it was detected that there was the same clear population structure of Chinese domestic goat populations, comprising four major genetic distinct groups, the Southwest China, the South China, the East China and the Northwest Chinas. The analysis of PCA was also consistent to the result of clustering, suggesting that the clustering of these Chinese goat populations was efficient and reliable in this experiment. In the studies of Zhang et al, the Nanjiang Yellow goat and the Leizhou goat in South China had also been clustered into one group, whereas the other three breeds in North China had been clustered into one group, consisting to our results [33]. Furthermore, it was observed that these goat populations had not been clustered by their purpose by the artificial selection, such as meat, cashmere or fur, on the contrary, the genetic relationships among the populations of goat reflected the natural geographical locations of the populations. The cause of the results here might be due to the short time of artificial selection in China. We also found that the populations with the close geographical distance were characterized by low genetic differentiation, but as the distance among the populations had been increased, the genetic differentiation among the populations also increased. Similarly, the results of AMOVA also supported the observed patterns of population differentiation, which indicated significant molecular variance both among populations (13.15%) and among individuals within populations (9.12%) (Table 3).

In the three clustering methods, we found that the current genetic structure of Chinese goats has resulted from the special geographical structure, especially in the Western China. It was observed that the Hengduan Mountains located at the middle of Qinghai-Tibet Plateau and Yunnan-Guizhou Plateau, which restricted the gene flow between XZS in Tibet and other goat populations (such as LLS, YLS, ZTS and JCS) in the Yunnan province.

In addition, as a geographical boundary in China, the Qinling Mountains-Huaihe River Line which divides the regions of China into the South and the North, and generates the difference between the South and the North: the climate, agricultural forms, geographical features as well as the human’s habits. In the present study, our results clearly demonstrated that the Qinling Mountains-Huaihe River Line also had separated the West goat populations into two clusters: the Southwest cluster and the Northwest cluster. Although with some admixture to the Northwest China population, our results indicated that the LNS located in the north of China had been clustered into the Southwest China populations, inconsistent to other results [33]. The reasons caused the difference are following: firstly, there is 30 microsatellite loci selected for the analysis in our study, not the limit numbers; on the other hand, the number of goat populations in the genetic structure analysis is 40, sampling from almost all of the geographical regions, from high latitude to plains.

### The Origin of Goat in China

Previous genetic studies and archaeological data had revealed goats were probably first domesticated in the Fertile Crescent region of Near East around 10,000 years ago [1], [2]. Some studies hint that a second independent domestication in Pakistan had given rise to the Cashmere-like goat breeds [1], [34]. However, the question that where is the Chinese goats from, has not been elaborated completely, although there are so many studies focus on it [8], [35], [36], [37]. Phylogenetic analysis of the first hypervariable region of mitochondrial DNA control region had suggested that the goats has multiple maternal origins with a possible center of origin in Aisa, as well as in the Fertile Crescent [8]. Li et al studied the mtDNA RFLP of 18 native goat populations in China, indicating that the native goat populations in China might originate from two different maternal ancestors [35]. In addition, it was proven that at least one subclade of lineage B was originated from eastern Asia, especially in southwest China [36]. Some studies also support the hypothesis that the southwest of China might be one of the domestication centers [37], [38].

In our study, we estimated that the goat populations in Southwest China might be the earliest domestic goats in China, even in Asia. The reasons contributed to the result are as following. Firstly, the results of genetic structure analysis by STRUCTURE demonstrated that, the Northwest China populations combining with the Southwest populations had been clustered into the West populations, when K was 2. However, the East China populations had been clustered into a single clade. Similar result was also observed in the NJ-tree. In other words, if the goat populations in Southwest China were not the earliest domestic goats, the Northwest China populations would be an independent cluster, while the Southwest China and East China populations would be another cluster, however, it is inconsistent with the present results. The observation that the genetic diversity of Northwest China populations was higher than the Southwest and the East China populations provided additional evidence to support this hypothesis.

Secondly, the domestication process of livestock is closely associated to the human activities [5]. It is generally recognized that the modern humans in East Asia have a common origin in Africa, and the first entry of modern humans into the southern part of East Asia occurred about 18,000–60,000 years ago and was followed by a north-ward migration that coincided with the receding glaciers in that area [39], [40]. The evidence of archaeology also indicated that the modern Chinese humans were originated in the South China [41]. Therefore, the migratory routes of modern humans in China help us to better understand the domestication process of the goats.

Thirdly, the Southwest China has the most complex river systems and a profoundly complex and dynamic geological history, and it also is rich of biodiversity [16], [17]. The special geographic structures of the region, such as the Hengduan Mountains and Qingling-Huaihe River Line, had restricted the gene flow of the livestock between the North and Southwest China goat populations. In this region, it is appear that multiple origin events documented as common phenomena in domestic animals such as cattle, chickens and pigs, suggesting that the southwest of China might be one of the domestication centers [42], [43], [44], [45]. Moreover, the mitochondrial DNA analysis of the goats in five Asian countries around of China (India, Pakistan, Mongolia, Malaysia and Laos) had revealed that the mtDNA lineage B were higher in Chinese goats than those of mixed goat populations in the five countries, suggesting that mtDNA lineage B of goats probably originated from China [37]. Combing with our results, it is inferred that the goat populations in Southwest China might be the earliest domestic goats in China, even in Asia.

Certainly, the results of microsatellite markers don’t have the convincing evidence to support the hypothesis completely, due to the low density of microsatellite markers in the DNA nuclear. Therefore, it is necessary to explore the origin of Chinese goats through a detailed study of goats in Asia using the archeological records, ancient DNA, wild goat samples from more locations, and more accurate molecular methods, such as the genome-wide SNP array and mitochondrial DNA.

In this study, we had analyzed the diversity of forty Chinese domestic goat breeds which were come from all the regions of China, from Qinghai-Tibet plateau region to the north and south agricultural region, and these breeds also had special purpose, such as meat, cashmere, fur and so on. The results here provide valuable information to help understanding the genetic structure of these important indigenous goats for future genetic improvement and setting the conservation program in China. The information of this study also would help to formulate the species-specific breeding programs to maintain the genetic characteristics and to reduce unnecessary inbreeding or gene flow among populations. The present work presents the first substantial analysis of the diversity of Chinese domestic goat microsatellite markers and provides information about the genetic structure of goat breeds within this important region, and thus insights into their genetic history. With the introduction of foreign species, these indigenous goat populations have been threatened, some of which have nearly disappeared or admixed with the exotic species. Assessment of the genetic status of Chinese indigenous goat population is essential for the establishment of the conservation programs.

## Supporting Information

Table S1 **The information of the 40 Chinese indigenous goat populations.** The information included the name, number and phenotypic characteristic of these goat populations, even the geographical location in China.
(DOC)

Table S2 **Bottleneck analysis of the 40 Chinese indigenous goat populations.**
(DOC)

Table S3 **Pairwise F_ST_ values (upper diagonal) and D_A_ genetic distance (lower diagonal) between 40 goat populations.**
(DOC)
