# Supplementary material for: Genetic Structure of Chinese Indigenous Goats and the Special Geographical Structure in the Southwest China as a Geographic Barrier Driving the Fragmentation of a Large Population
Source: PLoS One. 2014 Apr 9;9(4):e94435. doi: 10.1371/journal.pone.0094435 (PMC3981790; doi:10.1371/journal.pone.0094435)
Supplement: Table S1 — The information of the 40 Chinese indigenous goat populations. The information included the name, number and phenotypic characteristic of these goat populations, even the geographical location in China. (DOC) [file pone.0094435.s001.doc]

**Table S1: The information of the 40 Chinese indigenous goat populations.**

|  |  |  |  |  |  |
| --- | --- | --- | --- | --- | --- |
| **No.** | **Population** | **Abbreviation** | **Number** | **Phenotypic descriptors** | **Location** |
| 1 | Longling goat | LLS | 52 | Meat | Yunan |
| 2 | Maguan poll goat | MGS | 50 | Meat | Yunan |
| 3 | Yuling goat | YLS | 47 | Meat, fur | Yunan |
| 4 | Zhaotong goat | ZTS | 50 | Meat, fur | Yunan |
| 5 | Jianchang black goat | JCS | 60 | Meat, fur | Sichuan |
| 6 | Guishan goat | GSS | 59 | Milk, meat | Yunan |
| 7 | Fengqing Poll Black goat | FQH | 43 | Meat | Yunan |
| 8 | Guizhou White goat | GZS | 50 | Meat | Guizhou |
| 9 | Liaoning Cashmere goat | LNS | 58 | Cashmere, meat | Liaoning |
| 10 | Chengdu Brown goat | CDM | 58 | Meat, fur | Sichuan |
| 11 | Gulin Ma goat | GLM | 60 | Meat, fur | Sichuan |
| 12 | Longlin goat | LLY | 52 | Meat | Guangxi |
| 13 | Leizhou goat | LZS | 56 | Meat | Guangdong |
| 14 | Hainan East goat | HND | 58 | Wool, meat | Hainan |
| 15 | Du’an goat | DAS | 36 | Meat | Guangxi |
| 16 | Inner Mongolia Cashmere goat | MGR | 48 | Cashmere, meat | Inner Mongolia |
| 17 | Chaidamu goat | CDS | 59 | Cashmere | Qinhai |
| 18 | Xinjiang goat | XJS | 59 | Cashmere, meat | Xinjiang |
| 19 | Xizang goat (Tibetan goat) | XZS | 53 | Meat, Cashmere, fur | Xizang |
| 20 | Hexi Cashmere goat | HXR | 43 | Cashmere, meat | Gansu |
| 21 | Zhongwei goat | ZWS | 25 | Fur | Ningxia |
| 22 | Shannan White goat | SNB | 56 | Meat, fur | Shaanxi |
| 23 | Banjiao goat | BJS | 58 | Meat, fur | Sichuan |
| 24 | Funiu White goat | FNB | 45 | Meat, fur | Henan |
| 25 | Henan Niutui goat | HNN | 60 | Meat, fur | Henan |
| 26 | Huanghuai goat | HWS | 50 | Fur, meat | Henan |
| 27 | Jining Gray goat | JNQ | 59 | Fur | Shandong |
| 28 | Yimeng Black goat | YMH | 53 | Meat | Shandong |
| 29 | Lubei White goat | LBB | 48 | Meat, fur | Shandong |
| 30 | Lvliang Black goat | LLH | 58 | Meat, Cashmere | Shanxi |
| 31 | Taihang goat | THS | 60 | Meat, Cashmere | Shanxi |
| 32 | Yangtse River Delta White goat | CJB | 42 | Wool | Jiangsu |
| 33 | Chuandong White goat | CDB | 60 | Meat, fur | Chongqin |
| 34 | Guangfeng goat | GFS | 44 | Meat | Jiangxi |
| 35 | Ganxi goat | GXS | 43 | Meat | Jiangxi |
| 36 | Matou goat | MTS | 60 | Meat, fur | Hunan |
| 37 | Yichang White goat | YCB | 52 | Meat, fur | Hubei |
| 38 | Xiangdong Black goat | XDH | 52 | Fur, meat | Hunan |
| 39 | Fuqing goat | FQS | 55 | Meat | Fujian |
| 40 | Daiyun goat | DYS | 47 | Meat | Fujian |
