# Supplementary material for: Genetic Structure of Chinese Indigenous Goats and the Special Geographical Structure in the Southwest China as a Geographic Barrier Driving the Fragmentation of a Large Population
Source: PLoS One. 2014 Apr 9;9(4):e94435. doi: 10.1371/journal.pone.0094435 (PMC3981790; doi:10.1371/journal.pone.0094435)
Supplement: Table S2 — Bottleneck analysis of the 40 Chinese indigenous goat populations. (DOC) [file pone.0094435.s002.doc]

**Table S2. Bottleneck analysis of the 40 Chinese indigenous goat populations.**

|  |  |  |  |  |  |
| --- | --- | --- | --- | --- | --- |
| **Number** | **Population** | **IAM** | **TPM** | **SMM** | **Mode-shift** |
| 1 | LLS | 0.00000 | 0.00000 | 0.00012 | Shifted mode |
| 2 | MGS | 0.00000 | 0.00003 | 0.00017 | Shifted mode |
| 3 | YLS | 0.00000 | 0.00016 | 0.00138 | Shifted mode |
| 4 | ZTS | 0.00000 | 0.00407 | 0.01239 | Normal L-shaped |
| 5 | JCS | 0.00000 | 0.00000 | 0.00002 | Shifted mode |
| 6 | GSS | 0.00000 | 0.00033 | 0.01311 | Normal L-shaped |
| 7 | FQH | 0.00000 | 0.00019 | 0.00036 | Shifted mode |
| 8 | GZS | 0.00000 | 0.00025 | 0.00269 | Normal L-shaped |
| 9 | LNS | 0.00000 | 0.00001 | 0.00025 | Shifted mode |
| 10 | CDM | 0.00000 | 0.00000 | 0.00004 | Shifted mode |
| 11 | GLM | 0.00000 | 0.00269 | 0.04597 | Normal L-shaped |
| 12 | LLY | 0.00000 | 0.00004 | 0.00233 | Shifted mode |
| 13 | LZS | 0.00000 | 0.00000 | 0.00010 | Shifted mode |
| 14 | HND | 0.00000 | 0.00000 | 0.00000 | Shifted mode |
| 15 | DAS | 0.00000 | 0.00000 | 0.00000 | Shifted mode |
| 16 | MGR | 0.00000 | 0.00028 | 0.00983 | Shifted mode |
| 17 | CDS | 0.00000 | 0.00356 | 0.06468 | Normal L-shaped |
| 18 | XJS | 0.00000 | 0.00101 | 0.06207 | Normal L-shaped |
| 19 | XZS | 0.00000 | 0.00004 | 0.00310 | Shifted mode |
| 20 | HXR | 0.00000 | 0.00086 | 0.00642 | Shifted mode |
| 21 | ZWS | 0.00000 | 0.00174 | 0.01311 | Shifted mode |
| 22 | SNB | 0.00000 | 0.00000 | 0.00002 | Shifted mode |
| 23 | BJS | 0.00000 | 0.00289 | 0.07300 | Normal L-shaped |
| 24 | FNB | 0.00000 | 0.00067 | 0.01822 | Shifted mode |
| 25 | HNN | 0.00000 | 0.00013 | 0.01105 | Normal L-shaped |
| 26 | HWS | 0.00000 | 0.00057 | 0.00356 | Normal L-shaped |
| 27 | JNQ | 0.00000 | 0.00000 | 0.00062 | Normal L-shaped |
| 28 | YMH | 0.00000 | 0.00822 | 0.14942 | Normal L-shaped |
| 29 | LBB | 0.00000 | 0.00079 | 0.02133 | Normal L-shaped |
| 30 | LLH | 0.00000 | 0.00356 | 0.02245 | Normal L-shaped |
| 31 | THS | 0.00000 | 0.00044 | 0.00530 | Shifted mode |
| 32 | CJB | 0.00000 | 0.00000 | 0.00001 | Shifted mode |
| 33 | CDB | 0.00000 | 0.00021 | 0.00530 | Normal L-shaped |
| 34 | GFS | 0.00000 | 0.00002 | 0.00036 | Shifted mode |
| 35 | GXS | 0.00000 | 0.00079 | 0.00873 | Shifted mode |
| 36 | MTS | 0.00000 | 0.00000 | 0.00008 | Shifted mode |
| 37 | YCB | 0.00000 | 0.00005 | 0.00202 | Shifted mode |
| 38 | XDH | 0.00000 | 0.00000 | 0.00007 | Shifted mode |
| 39 | FQS | 0.00000 | 0.00000 | 0.00001 | Shifted mode |
| 40 | DYS | 0.00000 | 0.00000 | 0.00000 | Shifted mode |
