# Supplementary material for: Genetic Structure of Chinese Indigenous Goats and the Special Geographical Structure in the Southwest China as a Geographic Barrier Driving the Fragmentation of a Large Population
Source: PLoS One. 2014 Apr 9;9(4):e94435. doi: 10.1371/journal.pone.0094435 (PMC3981790; doi:10.1371/journal.pone.0094435)
Supplement: Table S3 — Pairwise FST values (upper diagonal) and DA genetic distance (lower diagonal) between 40 goat populations. (DOC) [file pone.0094435.s003.doc]

**Table S3: Pairwise F_ST_ values (upper diagonal) and D_A_ genetic distance (lower diagonal) between 40 goat populations.**

|  | LLS | MGS | YLS | ZTS | JCS | GSS | FQH | GZS | LNS | CDM | GLM | LLY | LZS | HND | DAS | MGR | CDS | XJS | XZS | HXR | ZWS | SNB | BJS | FNB | HNN | HWS | JNQ | YMH | LBB | LLH | THS | CJB | CDB | GFS | GXS | MTS | YCB | XDH | FQS | DYS |
| --- | --- | --- | --- | --- | --- | --- | --- | --- | --- | --- | --- | --- | --- | --- | --- | --- | --- | --- | --- | --- | --- | --- | --- | --- | --- | --- | --- | --- | --- | --- | --- | --- | --- | --- | --- | --- | --- | --- | --- | --- |
| LLS | - | 0.09259 | 0.04703 | 0.048 | 0.0754 | 0.06278 | 0.15255 | 0.15065 | 0.16896 | 0.21387 | 0.1414 | 0.12247 | 0.25124 | 0.21986 | 0.17885 | 0.21336 | 0.14844 | 0.16037 | 0.18088 | 0.15633 | 0.17091 | 0.17397 | 0.16933 | 0.14188 | 0.14892 | 0.15949 | 0.17323 | 0.13167 | 0.19615 | 0.17571 | 0.19779 | 0.20247 | 0.20335 | 0.26724 | 0.25208 | 0.21329 | 0.19953 | 0.22444 | 0.2786 | 0.33814 |
| MGS | 0.138 | - | 0.05165 | 0.06725 | 0.10203 | 0.09232 | 0.18778 | 0.13696 | 0.16312 | 0.22414 | 0.14135 | 0.13691 | 0.21687 | 0.19279 | 0.1569 | 0.23312 | 0.16425 | 0.16866 | 0.19113 | 0.17595 | 0.18034 | 0.18068 | 0.15428 | 0.1356 | 0.17043 | 0.15595 | 0.16973 | 0.13189 | 0.18252 | 0.17127 | 0.20234 | 0.19757 | 0.1996 | 0.2477 | 0.2405 | 0.20126 | 0.18741 | 0.20392 | 0.24423 | 0.32109 |
| YLS | 0.093 | 0.092 | - | 0.02246^NS^ | 0.03343 | 0.04746 | 0.12951 | 0.10524 | 0.15091 | 0.18336 | 0.11347 | 0.12005 | 0.21681 | 0.17877 | 0.1432 | 0.18681 | 0.12495 | 0.13483 | 0.15188 | 0.12696 | 0.14797 | 0.14773 | 0.14476 | 0.11044 | 0.12915 | 0.12648 | 0.14069 | 0.10677 | 0.15394 | 0.13803 | 0.17148 | 0.16998 | 0.17179 | 0.20968 | 0.20617 | 0.1777 | 0.15652 | 0.17725 | 0.23702 | 0.29343 |
| ZTS | 0.109 | 0.12 | 0.065 | - | 0.05892 | 0.05939 | 0.15282 | 0.11626 | 0.16549 | 0.20248 | 0.13603 | 0.12992 | 0.23273 | 0.20055 | 0.16869 | 0.21624 | 0.15527 | 0.15243 | 0.16913 | 0.15613 | 0.1733 | 0.17083 | 0.15835 | 0.13514 | 0.15526 | 0.14378 | 0.16558 | 0.1304 | 0.18759 | 0.16518 | 0.19994 | 0.18913 | 0.19511 | 0.24428 | 0.22793 | 0.19822 | 0.1818 | 0.20918 | 0.26223 | 0.33047 |
| JCS | 0.118 | 0.136 | 0.071 | 0.087 | - | 0.07355 | 0.1479 | 0.13744 | 0.17989 | 0.21072 | 0.12679 | 0.14907 | 0.24313 | 0.20258 | 0.15962 | 0.19048 | 0.13543 | 0.14907 | 0.16644 | 0.13976 | 0.15869 | 0.16125 | 0.16097 | 0.12265 | 0.15432 | 0.14577 | 0.15558 | 0.12665 | 0.18303 | 0.16427 | 0.19216 | 0.19674 | 0.17781 | 0.23824 | 0.24401 | 0.19525 | 0.17125 | 0.19099 | 0.2582 | 0.30725 |
| GSS | 0.116 | 0.172 | 0.129 | 0.15 | 0.161 | - | 0.07555 | 0.0988 | 0.10851 | 0.15475 | 0.0824 | 0.08615 | 0.15596 | 0.14619 | 0.11436 | 0.1287 | 0.06353 | 0.07437 | 0.09443 | 0.06651 | 0.0773 | 0.09005 | 0.10643 | 0.06017 | 0.06712 | 0.07305 | 0.0909 | 0.06063 | 0.10399 | 0.09504 | 0.11989 | 0.13161 | 0.12709 | 0.16409 | 0.16766 | 0.14343 | 0.12622 | 0.1496 | 0.19441 | 0.23604 |
| FQH | 0.214 | 0.262 | 0.215 | 0.238 | 0.228 | 0.158 | - | 0.2171 | 0.23032 | 0.2307 | 0.11252 | 0.11815 | 0.20589 | 0.21008 | 0.18807 | 0.20304 | 0.11252 | 0.12659 | 0.16006 | 0.1325 | 0.1501 | 0.13987 | 0.16786 | 0.13551 | 0.12118 | 0.13047 | 0.13951 | 0.10108 | 0.16049 | 0.15495 | 0.1806 | 0.18859 | 0.16863 | 0.22893 | 0.24227 | 0.19406 | 0.18238 | 0.21475 | 0.26114 | 0.29243 |
| GZS | 0.189 | 0.177 | 0.156 | 0.163 | 0.184 | 0.174 | 0.304 | - | 0.10873 | 0.21146 | 0.16251 | 0.18799 | 0.21034 | 0.16976 | 0.14007 | 0.21863 | 0.15766 | 0.16105 | 0.18054 | 0.15483 | 0.17312 | 0.15676 | 0.14768 | 0.13142 | 0.1632 | 0.13607 | 0.14707 | 0.15442 | 0.18201 | 0.17812 | 0.19576 | 0.18514 | 0.16495 | 0.21087 | 0.22162 | 0.17369 | 0.15407 | 0.17978 | 0.22763 | 0.25746 |
| LNS | 0.222 | 0.247 | 0.24 | 0.25 | 0.275 | 0.179 | 0.35 | 0.153 | - | 0.24609 | 0.18097 | 0.18475 | 0.21374 | 0.17323 | 0.15255 | 0.16162 | 0.10988 | 0.12211 | 0.16748 | 0.11808 | 0.12723 | 0.12337 | 0.14282 | 0.09987 | 0.12474 | 0.11131 | 0.12292 | 0.12794 | 0.13972 | 0.14287 | 0.14352 | 0.14608 | 0.16872 | 0.22484 | 0.2128 | 0.16602 | 0.15061 | 0.18244 | 0.20231 | 0.24236 |
| CDM | 0.282 | 0.306 | 0.26 | 0.291 | 0.288 | 0.237 | 0.312 | 0.276 | 0.333 | - | 0.12102 | 0.12243 | 0.19485 | 0.17616 | 0.17646 | 0.20733 | 0.17408 | 0.15339 | 0.17303 | 0.16084 | 0.17267 | 0.16202 | 0.15922 | 0.14862 | 0.12769 | 0.13546 | 0.13698 | 0.14536 | 0.15332 | 0.17716 | 0.18819 | 0.1824 | 0.18608 | 0.24047 | 0.18874 | 0.1866 | 0.1591 | 0.19844 | 0.2469 | 0.31708 |
| GLM | 0.219 | 0.22 | 0.197 | 0.225 | 0.212 | 0.176 | 0.195 | 0.226 | 0.298 | 0.16 | - | 0.05616 | 0.12621 | 0.14853 | 0.11986 | 0.15507 | 0.09613 | 0.09711 | 0.12759 | 0.09795 | 0.11167 | 0.10607 | 0.11128 | 0.08574 | 0.08113 | 0.07967 | 0.09244 | 0.06375 | 0.1173 | 0.10368 | 0.1303 | 0.13277 | 0.11746 | 0.16189 | 0.15389 | 0.1301 | 0.11259 | 0.13519 | 0.19562 | 0.23986 |
| LLY | 0.194 | 0.216 | 0.197 | 0.22 | 0.226 | 0.184 | 0.206 | 0.257 | 0.292 | 0.162 | 0.116 | - | 0.13203 | 0.14821 | 0.13878 | 0.17661 | 0.10833 | 0.09543 | 0.13447 | 0.1115 | 0.12081 | 0.11918 | 0.11897 | 0.10076 | 0.08682 | 0.09487 | 0.11557 | 0.07511 | 0.12325 | 0.12873 | 0.14211 | 0.14622 | 0.14394 | 0.21174 | 0.17485 | 0.159 | 0.14256 | 0.1693 | 0.21073 | 0.27088 |
| LZS | 0.306 | 0.262 | 0.281 | 0.31 | 0.324 | 0.242 | 0.275 | 0.262 | 0.296 | 0.265 | 0.175 | 0.175 | - | 0.09724 | 0.0969 | 0.23121 | 0.15155 | 0.1514 | 0.19427 | 0.16892 | 0.17919 | 0.15159 | 0.14546 | 0.14892 | 0.13139 | 0.11332 | 0.12169 | 0.10508 | 0.12702 | 0.15146 | 0.17621 | 0.1439 | 0.16799 | 0.21091 | 0.19536 | 0.16723 | 0.14876 | 0.16869 | 0.17472 | 0.22957 |
| HND | 0.255 | 0.23 | 0.225 | 0.246 | 0.264 | 0.211 | 0.267 | 0.224 | 0.233 | 0.224 | 0.187 | 0.182 | 0.133 | - | 0.05705 | 0.21597 | 0.13959 | 0.14381 | 0.19512 | 0.16009 | 0.17811 | 0.12792 | 0.13804 | 0.15801 | 0.13689 | 0.12619 | 0.13323 | 0.14992 | 0.1549 | 0.17498 | 0.18226 | 0.15087 | 0.16756 | 0.20726 | 0.17763 | 0.16018 | 0.13688 | 0.13972 | 0.16158 | 0.2038 |
| DAS | 0.24 | 0.212 | 0.228 | 0.251 | 0.244 | 0.2 | 0.274 | 0.224 | 0.253 | 0.266 | 0.195 | 0.21 | 0.169 | 0.115 | - | 0.17575 | 0.11107 | 0.12183 | 0.14111 | 0.12485 | 0.14364 | 0.11871 | 0.10931 | 0.11361 | 0.1149 | 0.1008 | 0.10297 | 0.11537 | 0.13682 | 0.15205 | 0.15536 | 0.13101 | 0.12981 | 0.18255 | 0.1534 | 0.13627 | 0.11948 | 0.11381 | 0.13956 | 0.1658 |
| MGR | 0.297 | 0.346 | 0.318 | 0.325 | 0.324 | 0.216 | 0.305 | 0.32 | 0.289 | 0.298 | 0.263 | 0.273 | 0.319 | 0.302 | 0.287 | - | 0.09068 | 0.08938 | 0.11039 | 0.06539 | 0.08704 | 0.11641 | 0.1485 | 0.09763 | 0.09688 | 0.12109 | 0.11109 | 0.11684 | 0.13961 | 0.14646 | 0.159 | 0.18701 | 0.17377 | 0.24016 | 0.22224 | 0.18491 | 0.16135 | 0.18805 | 0.23998 | 0.27608 |
| CDS | 0.23 | 0.267 | 0.246 | 0.273 | 0.258 | 0.135 | 0.211 | 0.264 | 0.21 | 0.277 | 0.212 | 0.217 | 0.262 | 0.23 | 0.231 | 0.174 | - | 0.02859^NS^ | 0.05616 | 0.02377 | 0.03234 | 0.04825 | 0.0908 | 0.05859 | 0.05977 | 0.06951 | 0.07 | 0.05959 | 0.08676 | 0.08588 | 0.09669 | 0.11717 | 0.12113 | 0.15924 | 0.15248 | 0.12681 | 0.1022 | 0.13556 | 0.16789 | 0.20675 |
| XJS | 0.241 | 0.264 | 0.255 | 0.263 | 0.267 | 0.141 | 0.226 | 0.259 | 0.215 | 0.251 | 0.209 | 0.203 | 0.252 | 0.23 | 0.238 | 0.149 | 0.09 | - | 0.06032 | 0.01863 | 0.02444 | 0.05574 | 0.09801 | 0.06175 | 0.06573 | 0.06909 | 0.07703 | 0.06429 | 0.08972 | 0.09284 | 0.10148 | 0.10923 | 0.12487 | 0.17428 | 0.1527 | 0.13463 | 0.11298 | 0.1444 | 0.17437 | 0.23072 |
| XZS | 0.258 | 0.283 | 0.271 | 0.273 | 0.28 | 0.174 | 0.266 | 0.281 | 0.26 | 0.283 | 0.245 | 0.229 | 0.286 | 0.284 | 0.254 | 0.202 | 0.116 | 0.126 | - | 0.05522 | 0.06962 | 0.08132 | 0.12503 | 0.08471 | 0.08317 | 0.09162 | 0.08658 | 0.0889 | 0.11024 | 0.12529 | 0.13462 | 0.14962 | 0.14433 | 0.18358 | 0.18014 | 0.15517 | 0.13346 | 0.16168 | 0.1975 | 0.25628 |
| HXR | 0.246 | 0.292 | 0.258 | 0.282 | 0.269 | 0.141 | 0.239 | 0.265 | 0.223 | 0.262 | 0.221 | 0.225 | 0.279 | 0.25 | 0.255 | 0.149 | 0.086 | 0.08 | 0.127 | - | 0.0193^NS^ | 0.05427 | 0.08518 | 0.04661 | 0.05042 | 0.0605 | 0.0675 | 0.06191 | 0.08303 | 0.08181 | 0.08902 | 0.11657 | 0.12293 | 0.16682 | 0.15131 | 0.12871 | 0.10215 | 0.1317 | 0.16885 | 0.2183 |
| ZWS | 0.25 | 0.291 | 0.285 | 0.309 | 0.292 | 0.161 | 0.257 | 0.297 | 0.252 | 0.29 | 0.251 | 0.254 | 0.29 | 0.269 | 0.263 | 0.169 | 0.109 | 0.108 | 0.155 | 0.092 | - | 0.05412 | 0.09151 | 0.05302 | 0.0724 | 0.0739 | 0.08781 | 0.07326 | 0.10253 | 0.09217 | 0.10381 | 0.14695 | 0.14039 | 0.19407 | 0.17702 | 0.15079 | 0.12298 | 0.16789 | 0.20268 | 0.26211 |
| SNB | 0.256 | 0.277 | 0.263 | 0.273 | 0.279 | 0.172 | 0.23 | 0.259 | 0.226 | 0.263 | 0.222 | 0.216 | 0.252 | 0.194 | 0.228 | 0.209 | 0.14 | 0.151 | 0.165 | 0.156 | 0.16 | - | 0.0733 | 0.0802 | 0.06572 | 0.06881 | 0.06448 | 0.08255 | 0.08754 | 0.12003 | 0.11187 | 0.12116 | 0.12693 | 0.15316 | 0.15711 | 0.13281 | 0.10441 | 0.13926 | 0.1755 | 0.22521 |
| BJS | 0.228 | 0.226 | 0.224 | 0.24 | 0.242 | 0.192 | 0.264 | 0.209 | 0.236 | 0.225 | 0.181 | 0.194 | 0.225 | 0.182 | 0.19 | 0.246 | 0.189 | 0.197 | 0.228 | 0.19 | 0.204 | 0.144 | - | 0.08636 | 0.0858 | 0.07181 | 0.07702 | 0.0842 | 0.10193 | 0.11195 | 0.10899 | 0.12583 | 0.10599 | 0.1679 | 0.137 | 0.11947 | 0.09089 | 0.11559 | 0.17304 | 0.21335 |
| FNB | 0.217 | 0.251 | 0.23 | 0.252 | 0.24 | 0.125 | 0.229 | 0.227 | 0.204 | 0.25 | 0.187 | 0.204 | 0.249 | 0.242 | 0.225 | 0.197 | 0.141 | 0.138 | 0.165 | 0.135 | 0.145 | 0.164 | 0.179 | - | 0.04744 | 0.05001 | 0.05351 | 0.04538 | 0.07514 | 0.07211 | 0.09627 | 0.12449 | 0.10273 | 0.15282 | 0.1456 | 0.12562 | 0.09632 | 0.12171 | 0.1693 | 0.21331 |
| HNN | 0.229 | 0.286 | 0.247 | 0.275 | 0.283 | 0.138 | 0.225 | 0.268 | 0.215 | 0.207 | 0.186 | 0.181 | 0.229 | 0.209 | 0.23 | 0.193 | 0.139 | 0.139 | 0.164 | 0.126 | 0.164 | 0.133 | 0.17 | 0.104 | - | 0.04934 | 0.04872 | 0.0554 | 0.06741 | 0.08916 | 0.09352 | 0.10405 | 0.11044 | 0.15409 | 0.146 | 0.12511 | 0.10897 | 0.1232 | 0.15347 | 0.18814 |
| HWS | 0.269 | 0.272 | 0.263 | 0.273 | 0.281 | 0.169 | 0.25 | 0.238 | 0.22 | 0.224 | 0.177 | 0.2 | 0.223 | 0.204 | 0.223 | 0.228 | 0.161 | 0.15 | 0.182 | 0.151 | 0.178 | 0.15 | 0.152 | 0.119 | 0.125 | - | 0.03069 | 0.034 | 0.05774 | 0.08199 | 0.10171 | 0.09536 | 0.10042 | 0.14586 | 0.14471 | 0.12149 | 0.0885 | 0.12149 | 0.16486 | 0.19947 |
| JNQ | 0.288 | 0.278 | 0.269 | 0.288 | 0.291 | 0.182 | 0.252 | 0.251 | 0.241 | 0.243 | 0.179 | 0.222 | 0.221 | 0.219 | 0.224 | 0.209 | 0.167 | 0.165 | 0.177 | 0.17 | 0.201 | 0.158 | 0.161 | 0.128 | 0.13 | 0.079 | - | 0.04447 | 0.04995 | 0.09554 | 0.10578 | 0.09684 | 0.10262 | 0.15759 | 0.15036 | 0.12299 | 0.09424 | 0.12327 | 0.16148 | 0.18686 |
| YMH | 0.211 | 0.229 | 0.21 | 0.234 | 0.226 | 0.135 | 0.19 | 0.234 | 0.227 | 0.229 | 0.151 | 0.171 | 0.192 | 0.231 | 0.225 | 0.21 | 0.148 | 0.147 | 0.174 | 0.148 | 0.172 | 0.176 | 0.174 | 0.109 | 0.136 | 0.096 | 0.105 | - | 0.04654 | 0.05446 | 0.08333 | 0.08942 | 0.08857 | 0.1534 | 0.13624 | 0.10682 | 0.08486 | 0.11548 | 0.15398 | 0.19988 |
| LBB | 0.277 | 0.289 | 0.256 | 0.293 | 0.299 | 0.179 | 0.259 | 0.27 | 0.24 | 0.255 | 0.209 | 0.23 | 0.221 | 0.239 | 0.261 | 0.243 | 0.18 | 0.188 | 0.195 | 0.182 | 0.212 | 0.18 | 0.207 | 0.154 | 0.154 | 0.133 | 0.119 | 0.091 | - | 0.06681 | 0.08072 | 0.08047 | 0.1025 | 0.15464 | 0.13874 | 0.11233 | 0.09033 | 0.11746 | 0.14434 | 0.19634 |
| LLH | 0.272 | 0.276 | 0.253 | 0.27 | 0.282 | 0.19 | 0.269 | 0.268 | 0.247 | 0.281 | 0.203 | 0.238 | 0.232 | 0.261 | 0.284 | 0.265 | 0.186 | 0.204 | 0.221 | 0.194 | 0.217 | 0.242 | 0.218 | 0.173 | 0.203 | 0.183 | 0.192 | 0.124 | 0.128 | - | 0.02919 | 0.06882 | 0.07871 | 0.10277 | 0.0844 | 0.07359 | 0.05935 | 0.08773 | 0.12643 | 0.16644 |
| THS | 0.288 | 0.305 | 0.289 | 0.305 | 0.309 | 0.21 | 0.282 | 0.286 | 0.237 | 0.287 | 0.233 | 0.245 | 0.261 | 0.267 | 0.276 | 0.26 | 0.192 | 0.201 | 0.217 | 0.191 | 0.212 | 0.218 | 0.208 | 0.187 | 0.184 | 0.196 | 0.208 | 0.153 | 0.155 | 0.068 | - | 0.07551 | 0.09262 | 0.1033 | 0.07301 | 0.07321 | 0.06681 | 0.08784 | 0.12053 | 0.15325 |
| CJB | 0.299 | 0.31 | 0.289 | 0.308 | 0.32 | 0.232 | 0.3 | 0.276 | 0.237 | 0.283 | 0.241 | 0.256 | 0.225 | 0.243 | 0.253 | 0.308 | 0.222 | 0.203 | 0.243 | 0.229 | 0.262 | 0.235 | 0.231 | 0.232 | 0.21 | 0.203 | 0.223 | 0.176 | 0.176 | 0.133 | 0.128 | - | 0.09253 | 0.1215 | 0.11269 | 0.07584 | 0.0717 | 0.08897 | 0.10707 | 0.13098 |
| CDB | 0.266 | 0.269 | 0.265 | 0.284 | 0.281 | 0.213 | 0.262 | 0.23 | 0.246 | 0.25 | 0.187 | 0.221 | 0.23 | 0.225 | 0.228 | 0.27 | 0.218 | 0.217 | 0.251 | 0.223 | 0.244 | 0.213 | 0.157 | 0.197 | 0.196 | 0.186 | 0.195 | 0.155 | 0.177 | 0.126 | 0.129 | 0.141 | - | 0.11609 | 0.1082 | 0.07208 | 0.05542 | 0.07464 | 0.1328 | 0.13862 |
| GFS | 0.32 | 0.313 | 0.3 | 0.325 | 0.336 | 0.251 | 0.29 | 0.272 | 0.299 | 0.316 | 0.247 | 0.291 | 0.234 | 0.246 | 0.256 | 0.341 | 0.26 | 0.285 | 0.278 | 0.28 | 0.289 | 0.251 | 0.247 | 0.255 | 0.245 | 0.245 | 0.262 | 0.228 | 0.216 | 0.139 | 0.13 | 0.151 | 0.148 | - | 0.09859 | 0.10751 | 0.1043 | 0.1038 | 0.17683 | 0.17857 |
| GXS | 0.311 | 0.297 | 0.29 | 0.307 | 0.332 | 0.247 | 0.3 | 0.279 | 0.297 | 0.281 | 0.234 | 0.259 | 0.247 | 0.231 | 0.246 | 0.321 | 0.253 | 0.259 | 0.279 | 0.267 | 0.284 | 0.268 | 0.222 | 0.252 | 0.239 | 0.249 | 0.259 | 0.208 | 0.22 | 0.13 | 0.109 | 0.159 | 0.129 | 0.102 | - | 0.07128 | 0.07124 | 0.07989 | 0.1323 | 0.18199 |
| MTS | 0.293 | 0.29 | 0.285 | 0.291 | 0.31 | 0.237 | 0.277 | 0.252 | 0.272 | 0.27 | 0.216 | 0.258 | 0.243 | 0.228 | 0.268 | 0.29 | 0.236 | 0.237 | 0.265 | 0.247 | 0.274 | 0.236 | 0.202 | 0.242 | 0.223 | 0.225 | 0.239 | 0.189 | 0.196 | 0.134 | 0.129 | 0.133 | 0.106 | 0.142 | 0.116 | - | 0.01798^NS^ | 0.05013 | 0.08861 | 0.12403 |
| YCB | 0.279 | 0.273 | 0.268 | 0.278 | 0.287 | 0.235 | 0.279 | 0.233 | 0.25 | 0.257 | 0.207 | 0.248 | 0.236 | 0.209 | 0.251 | 0.285 | 0.224 | 0.23 | 0.25 | 0.224 | 0.252 | 0.212 | 0.166 | 0.216 | 0.223 | 0.184 | 0.206 | 0.167 | 0.179 | 0.13 | 0.133 | 0.152 | 0.094 | 0.153 | 0.13 | 0.057 | - | 0.03529 | 0.09518 | 0.13479 |
| XDH | 0.303 | 0.27 | 0.274 | 0.299 | 0.303 | 0.254 | 0.299 | 0.258 | 0.276 | 0.288 | 0.214 | 0.258 | 0.226 | 0.2 | 0.214 | 0.298 | 0.253 | 0.249 | 0.269 | 0.247 | 0.294 | 0.244 | 0.195 | 0.235 | 0.228 | 0.211 | 0.226 | 0.186 | 0.189 | 0.148 | 0.15 | 0.15 | 0.109 | 0.142 | 0.111 | 0.092 | 0.074 | - | 0.08041 | 0.12012 |
| FQS | 0.339 | 0.312 | 0.33 | 0.344 | 0.354 | 0.288 | 0.319 | 0.297 | 0.301 | 0.338 | 0.271 | 0.296 | 0.233 | 0.231 | 0.235 | 0.355 | 0.288 | 0.281 | 0.301 | 0.291 | 0.324 | 0.29 | 0.262 | 0.282 | 0.263 | 0.269 | 0.274 | 0.225 | 0.219 | 0.192 | 0.182 | 0.164 | 0.163 | 0.177 | 0.156 | 0.136 | 0.15 | 0.112 | - | 0.09671 |
| DYS | 0.378 | 0.359 | 0.379 | 0.399 | 0.393 | 0.315 | 0.336 | 0.308 | 0.331 | 0.37 | 0.295 | 0.333 | 0.27 | 0.258 | 0.246 | 0.377 | 0.316 | 0.329 | 0.349 | 0.33 | 0.355 | 0.337 | 0.296 | 0.314 | 0.283 | 0.299 | 0.29 | 0.266 | 0.276 | 0.219 | 0.206 | 0.183 | 0.171 | 0.182 | 0.177 | 0.162 | 0.191 | 0.15 | 0.112 | - |

The population abbreviations are shown in Table 1.

NS, all *P*-values applied to pairwise F_ST_ were significant at *P*<0.05 except those with NS for non-significant.
